# Acute cerebral ischemic stroke as a presentation of polycythemia vera: a case report and a review of literature

**DOI:** 10.1097/MS9.0000000000000582

**Published:** 2023-04-01

**Authors:** Mohamed Osman Siyad, Mohamed Farah Osman, Abdisamad Mohamed Adan, Mohamed Sheikh Hassan, Mohamed Osman Omar Jeele

**Affiliations:** a Department of Internal Medicine; b Department of Neurology, Mogadishu Somali Turkish Training and Research Hospital, Mogadishu, Somalia

**Keywords:** acute cerebral ischemia, basal ganglion infarct, polycythemia vera, stroke

## Abstract

Polycythemia vera (PV) is a disease of stem cells characterized by pan hyperplastic, malignant, and neoplastic bone marrow conditions. It is characterized by an increased absolute red blood cell count due to uncontrolled red blood cell synthesis, as well as excessive white blood cell and platelet production. Although the relationship between PV and stroke, especially ischemic stroke, is widely known around the world, no previous cases have been reported from Somalia.

**Case presentation:**

In the presenting study, we report a 60-year-old male patient who presented with a right-side weakness for 3 days. After laboratory and brain imaging, he was diagnosed with an acute cerebral infarct affecting the left basal ganglion secondary to PV.

**Conclusions:**

PV as the cause of ischemic stroke is a rare condition but can be encountered in clinical practice, and clinicians should be familiar with this combination.

## Introduction

Highlights

- Cerebral ischemia occurrences are triggered by elevated blood viscosity and platelet activation in the arteries of the central nervous system.
- Arterial ischemic consequences of polycythemia vera are widespread, typically occurring early in the disease cycle, spanning from 15% in the 2 years before diagnosis to up to 40% throughout the illness course.
- Polycythemia vera may be more commonly affected in the left hemisphere of the brain.

Polycythemia vera (PV) is the most prominent myeloproliferative neoplasm (MPN), the final phenotypic effect of *Janus kinase-2* (*JAK2*) somatic gene mutations, and the most frequently MPN accompanied by arterial and venous thrombosis^1^. It was first identified by a French doctor named Louis Henri Vaquez in 1892^2^. It is caused by signaling deficiency which will cause an aberrant response to growth stimuli, and the defective clonal line interferes with the proliferation of normal lineages. The intracellular signaling gene *JAK2* is mutated in 90% of PV cases^3^. It is characterized by an increased absolute red blood cell count (RBC) due to uncontrolled RBC synthesis, as well as excessive white blood cell (WBC) and platelet production^4^. The relationship between PV and stroke, especially ischemic stroke, is widely known around the world. There are numerous populations with a higher incidence of stroke; however, patients with PV are unique, both in terms of pathophysiology and the treatment of the disease^5^. Cerebral ischemia occurrences are triggered by elevated blood viscosity and platelet activation in the arteries of the central nervous system^6^. Here we report a 60-year-old male patient with ischemic stroke resulting from PV.

## Case presentation

**Figure 1:**
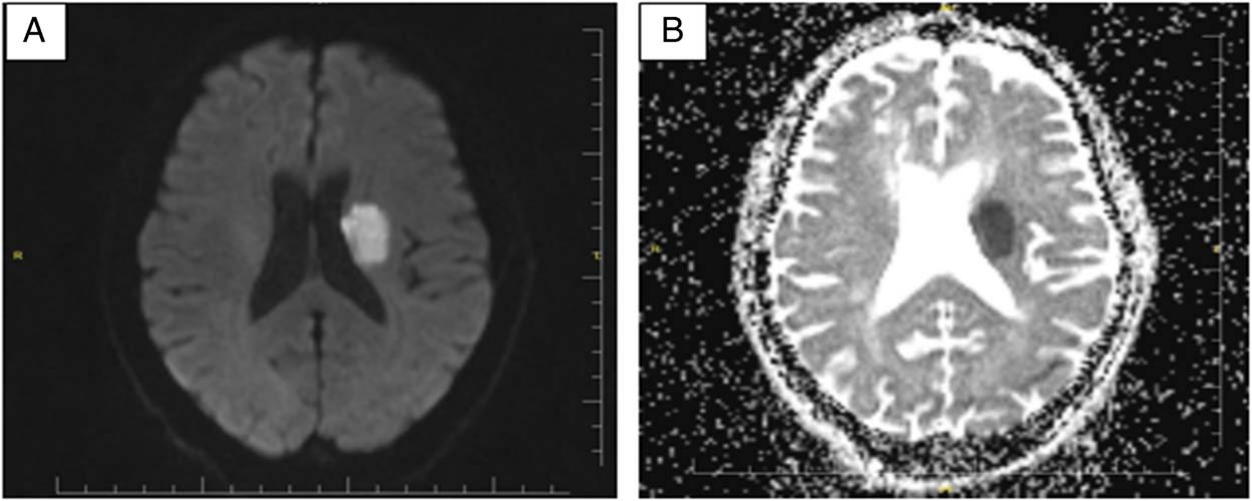
(A, B) Diffusion brain MRI showing diffusion restriction (hyperintensity in diffusion-weighted imaging and hypointensity in apparent diffusion coefficient) in the left basal ganglion consistent with an acute infarct.

A 60-year-old male was admitted from the emergency with a complaint of right-sided weakness for 3 days. Noncontrast brain computed tomography was inconclusive and did not reveal any focal lesion. Subsequent diffusion MRI showed left basal ganglion hyperintensity consistent with acute cerebral infarct (see Fig. 1). The patient had no history of cerebrovascular risk factors such as hypertension, hyperlipidemia, diabetes, coronary heart disease, or smoking. There was no recent history of trauma, poisoning, surgery, blood transfusion, infection, or a family history of a similar condition. There was no seizure, recent headache, or visual disturbance. Vital signs were normal. Despite basal ganglion infarct, the patient’s cognitive function was preserved. There was no apparent restriction of eye movements. Other systemic examinations, including those of the respiratory and cardiovascular systems, were unremarkable.

**Figure 2:**
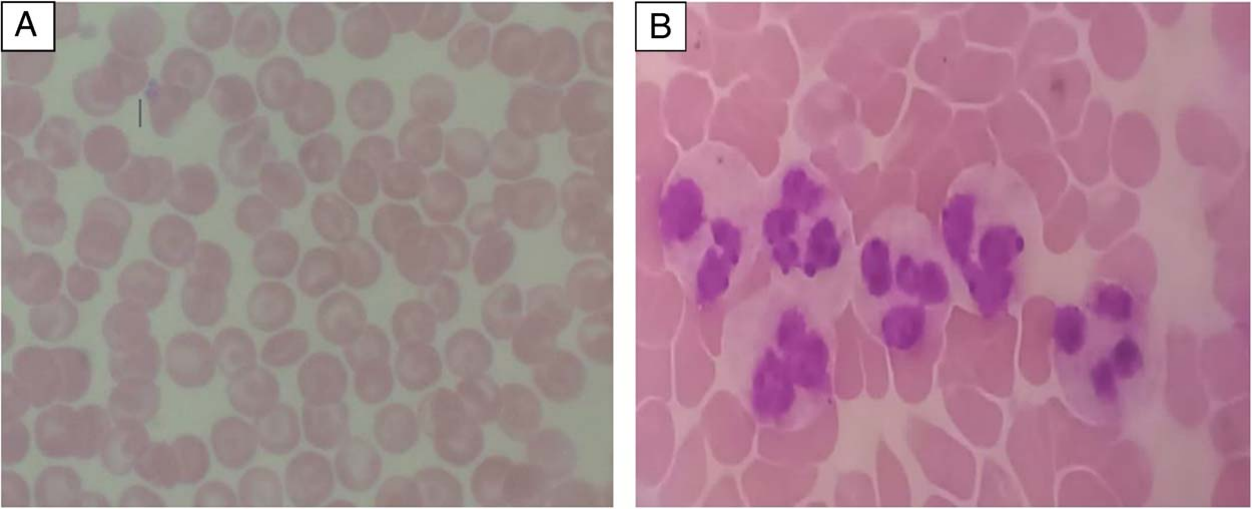
(A, B) A peripheral blood smear showed erythrocytosis and neutrophilia features suggestive of polycythemia.

The results of laboratory investigations on admission were as follows: whole blood cell analysis revealed WBC count, 37.09 ×10^9^/l; lymphocyte percentage, 2.5%; neutrophil percentage, 89.4%; monocyte absolute value, 1.10 ×10^9^/l; eosinophil absolute value, 0.3×10^9^/l; basophil absolute value, 0.02 ×10^9^/l; and basophil percentage, 0.1%. The RBC count was 9.89×10^12^/l; hemoglobin level, 21.3  g/dl; platelet count, 258×10^9^/l. The absolute value of neutrophils was 33.17×10^9^/l and the platelet volume was 9.7%. The liver function test, renal function test, glucose, and electrolytes were normal. There was no obvious abnormality among the results for the remaining blood sampling: abdominal ultrasonography showed no abnormalities. A 24-h dynamic electrocardiogram did not reveal atrial fibrillation or arrhythmia. Echocardiography did not show any potential cardioembolic source. Carotid and vertebral arteries Doppler ultrasound examination did not show intravascular thickening, thrombus, or stenosis. Peripheral blood smear showed erythrocytosis, and neutrophilia features suggestive of polycythemia (see Fig. 2).

Based on the case summary and the lack of capacity in our hospital to perform bone marrow aspiration and *JAK2* gene analysis, this patient’s cerebral infarction was diagnosed due to PV. The patient was treated with low molecular weight heparin 60 mg twice daily, aspirin at a dose of 100 mg, and hydroxyurea at a dose of 500 mg twice daily.

## Discussion

PV is a disease of stem cells characterized by pan hyperplastic, malignant, and neoplastic bone marrow conditions 7. According to 2016 revised WHO recommendations, the diagnosis of PV requires either the presence of all three major criteria or the presence of the first two major criteria and the minor criterion. The major WHO criteria include hemoglobin greater than 16.5 g/dl in men and greater than 16 g/dl in women, or hematocrit greater than 49% in men and greater than 48% in women, or red cell mass greater than 25% above mean normal predicted value, bone marrow biopsy demonstrating hypercellularity for age with trilineage growth (panmyelosis) consisting of prominent erythroid, granulocytic, and megakaryocytic proliferation with pleomorphic, and the presence of *JAK2V617F* or *JAK2* exon 12 mutations. A serum erythropoietin level below the normal range constitutes the minor criterion^8^. The peak incidence of PV is found between the ages of 50 and 70, correlating with the incidence rate of stroke at these ages^9^.

Arterial ischemic consequences of PV are widespread, typically occurring early in the disease cycle, spanning from 15% in the 2 years before diagnosis to up to 40% throughout the illness course^10^. The high hematocrit level, which is a determinant of blood hyperviscosity, may also initiate an attack on the arterial endothelium, equivalent to atheromatous lesions, leading to platelet activation and also activation of the inflammation response that contributes to arterial blockage and in situ thrombus formation^11^. In 20% of individuals with PV, thrombotic events are the presenting symptoms, and they are the leading cause of morbidity and mortality in untreated patients^12^.

To the extent of our knowledge, this is the first case of ischemic stroke caused by PV to be documented in Somalia. Our patient presented with right-sided weakness without a history of cerebrovascular risk factors such as hypertension, hyperlipidemia, diabetes, coronary heart disease, or smoking. His subsequent evaluations, which included blood testing and brain imaging, revealed acute cerebral infarction in the basal ganglion caused by PV. The patient was admitted to our hospital, and low molecular weight heparin, aspirin, and hydroxyurea were administered.

Gaye *et al*.^13^ described a case report of a 66-year-old male patient with recurrent ischemic stroke in 2022. They concluded that the cause of the recurrent ischemic stroke was identified as PV and was treated with hydroxyurea, aspirin, and allopurinol. Corse and Kurtis also described a case of a 51-year-old male patient in 2018 who presented with right-sided weakness and slurred speech. They diagnosed the patient with an ischemic stroke caused by secondary polycythemia due to erythropoietin-secreting renal cell carcinoma^14^. In 2013, Zoraster and Rison reported a case of a 57-year-old man with right-side clumsiness. PV was found to be the perpetrator of this condition, and the patient’s symptoms were relieved with hydration and phlebotomy^9^. As in our case report, all of the aforementioned cases presented to the hospital with right-side weakness, which could be interpreted as meaning that the left hemisphere is the most commonly affected hemisphere in PV cases. This needs to be the spotlight for future research to identify which hemisphere of the brain is most commonly affected by an ischemic stroke caused by PV.

Our work has been reported in line with the SCARE (Surgical CAse REport) 2020 criteria^15^.

## Conclusion

PV as the cause of ischemic stroke is a rare condition but can be encountered in clinical practice, and clinicians should be familiar with this combination. It seems that ischemic stroke patients with PV tend to be above the age of 50 and may always affect the left hemisphere of the brain.

## Ethical approval

Mogadishu Somali Turkish Training and Research Hospital ethics committee waived approval for this case report.

## Patient consent

Written informed consent was obtained from the patient for the publication of this case report. A copy of the written consent is available for review by the Editor-in-Chief of this journal on request.

## Sources of funding

The authors declare no funding source in relation to this work.

## Author contribution

All authors contributed substantially to this manuscript, whether in the conception, drafting, or revising of the final manuscript.

## Conflicts of interest disclosure

The authors declare no conflicts of interest.

## Research registration unique identifying number (UIN)

1. Name of the registry: not applicable.
2. Unique identifying number or registration ID: not applicable.
3. Hyperlink to your specific registration (must be publicly accessible and will be checked): not applicable.

## Guarantor

As a corresponding author, I, Mohamed Osman Omar Jeele, confirm that the manuscript has been read and approved by all named authors.

## Provenance and peer review

Not commissioned externally peer-reviewed.

## References

[1] Spivak JL . How I treat polycythemia vera. Blood 2019;134:341–352. 31151982 10.1182/blood.2018834044

[2] Vaquez H . Sur une forme speciale de cyanose s’accompanant d’hyperglobulie excessive et peristente (On a special form of cyanosis accompanied by excessive and persistent erythrocytosis). C R S Biol, Bull Mem Soc Med Hop Paris 1895;12:60; 1892;4:384–8.

[3] Barbui T Barosi G Birgegard G . European LeukemiaNet. Philadelphia-negative classical myeloproliferative neoplasms: critical concepts and management recommendations from European LeukemiaNet. J Clin Oncol 2011;29:761–770. 21205761 10.1200/JCO.2010.31.8436 PMC4979120

[4] Streiff MB Smith B Spivak JL . The diagnosis and management of polycythemia vera in the era since the Polycythemia Vera Study Group: a survey of American Society of Hematology members’ practice patterns. Blood 2002;99:1144–1149. 11830459 10.1182/blood.v99.4.1144

[5] Roger VL Go AS Lloyd-Jones DM . Heart disease and stroke statistics – 2011 update: a report from the American Heart Association. Circulation 2011;123:e18–e209. 21160056 10.1161/CIR.0b013e3182009701 PMC4418670

[6] Hart RG Kanter MC . Hematological disorders and ischemic stroke: a selective review. Stroke 1990;21:1111–1121. 2202092 10.1161/01.str.21.8.1111

[7] PDQ Adult Treatment Editorial Board. Chronic Myeloproliferative Neoplasms Treatment (PDQ®) PDQ Cancer Information Summaries. National Cancer Institute (US); 2020.

[8] Arber DA Orazi A Hasserjian R . The 2016 revision to the World Health Organization classification of myeloid neoplasms and acute leukemia. Blood 2016;127:2391–2405. 27069254 10.1182/blood-2016-03-643544

[9] Zoraster RM Rison RA . Acute embolic cerebral ischemia as an initial presentation of polycythemia vera: a case report. J Med Case Rep 2013;7:131. 23683307 10.1186/1752-1947-7-131 PMC3668271

[10] De Stefano V Za T Rossi E . Recurrent thrombosis in patients with polycythemia vera and essential thrombocythemia: incidence, risk factors, and effect of treatments. Haematologica 2008;93:372–380. 18268279 10.3324/haematol.12053

[11] Richter V Savery MD Gassmann M . Excessive erythrocytosis compromises the blood–endothelium interface in erythropoietin‐overexpressing mice. J Physiol 2011;589:5181–5192. 21859826 10.1113/jphysiol.2011.209262 PMC3225673

[12] Mowla A Shah H Lail NS . Successful intravenous thrombolysis for acute stroke caused by polycythemia vera. Arch Neurosci 2017;4:e62181.

[13] Gaye NM Toudou-Daouda M Chausson N . Recurrent ischemic stroke revealing polycythemia vera. Neurosci Med 2022;13:49–52.

[14] Corse AK Kurtis H . Ischemic stroke caused by secondary polycythemia and incidentally-found renal cell carcinoma: a case report. Am J Case Rep 2018;19:638. 29861497 10.12659/AJCR.909322 PMC6016561

[15] Agha RA Franchi T Sohrabi C . for the SCARE Group. The SCARE 2020 guideline: updating consensus Surgical CAse REport (SCARE) guidelines. Int J Surg 2020;84:226–230. 33181358 10.1016/j.ijsu.2020.10.034

